# Retropharyngeal Internal Carotid Artery Presenting as Chronic Otalgia and Throat Pain Mimicking Laryngopharyngeal Reflux: A Case Report

**DOI:** 10.7759/cureus.113031

**Published:** 2026-07-20

**Authors:** Katherine Rivero, Hanasoge Girishkumar, Frank Masella, Sania Thite, Reshma Pydi, Jigyasha Pradhan

**Affiliations:** 1 Department of Surgery, Bronx-Lebanon Hospital Center, Bronx, USA

**Keywords:** aberrant internal carotid artery, chronic otalgia, laryngopharyngeal reflux, pulsatile laryngeal mass, retropharyngeal internal carotid artery, throat & head neck surgery, throat pain, vascular anomaly

## Abstract

Retropharyngeal displacement of the internal carotid artery (ICA) is an uncommon anatomical variant with significant procedural safety implications. The vessel deviates medially into the retropharyngeal space, approaching or contacting the posterior pharyngeal mucosa, placing it at immediate risk of injury during routine pharyngeal instrumentation. We report a 54-year-old woman with type 2 diabetes mellitus, hypertension, and hyperlipidemia who experienced a three-year diagnostic delay before this anomaly was identified.

Her presentation of unilateral chronic otalgia and burning throat discomfort sequentially mimicked otitis externa and laryngopharyngeal reflux (LPR), both of which failed to respond to empirical treatment. CT of the neck with intravenous contrast revealed the right ICA approaching the pharyngeal mucosa. Flexible laryngoscopy demonstrated pulsatile submucosal bulging of the posterior pharyngeal wall, highly suggestive of an underlying vascular structure

Carotid duplex ultrasonography demonstrated no hemodynamically significant stenosis bilaterally. Conservative management was adopted, with provider notification initiated. At six-month follow-up, the patient remained neurologically intact with stable symptoms. This case highlights the importance of vascular anomaly recognition in patients with unexplained, treatment-refractory otolaryngologic symptoms and the potentially fatal consequences of unrecognized aberrant ICA during pharyngeal procedures.

## Introduction

The internal carotid artery (ICA) normally courses within the carotid space, lateral to the posterior pharyngeal wall and medial to the internal jugular vein. Retropharyngeal or aberrant ICA refers to a clinically important anatomical variant in which the vessel deviates medially, entering the retropharyngeal space and approaching or contacting the posterior oropharyngeal or hypopharyngeal mucosa. This anomaly may arise from congenital persistence of embryologic vascular patterns or from acquired arterial elongation driven by hypertension, atherosclerosis, and aging [1,2]. The clinical significance of this variant lies principally in procedural safety. When the aberrant ICA lies within millimeters of the pharyngeal mucosa, virtually any transoral intervention-including tonsillectomy, peritonsillar abscess drainage, transoral biopsy, adenoidectomy, and orotracheal intubation-carries a risk of catastrophic arterial hemorrhage [3,4]. Published case series have documented life-threatening ICA injuries during procedures that would be considered routine had the vascular anomaly been recognized preoperatively [5,6]. Equally important is the diagnostic challenge this variant poses. Symptoms including unilateral otalgia, throat pain, globus sensation, and dysphagia closely mimic common otolaryngologic conditions, often resulting in a protracted diagnostic delay [7,8]. Laryngopharyngeal reflux, otitis externa, eustachian tube dysfunction, and temporomandibular joint disorders are among the diagnoses most frequently attributed to these symptoms [9]. We report a case of symptomatic retropharyngeal ICA with a three-year diagnostic delay, a narrative review of previously reported cases, and propose a management and notification protocol applicable in community and academic practice.

## Case presentation

A 54-year-old woman with type 2 diabetes mellitus, hypertension, and hyperlipidemia presented with a three-year history of recurrent moderate right-sided otalgia, burning throat discomfort, and right submandibular pain that regularly disrupted sleep. Symptoms were strictly unilateral throughout the clinical course and were exacerbated by spicy or acidic foods. At initial presentation, symptoms were attributed to otitis externa, and topical treatment was initiated, yielding only transient partial relief; ear cultures obtained at that time were negative. A subsequent working diagnosis of laryngopharyngeal reflux prompted empirical therapy with a proton pump inhibitor (omeprazole 20 mg each morning) and an H2-receptor antagonist (famotidine 20 mg nightly) maintained for two months without symptomatic improvement. The patient denied dysphagia, odynophagia, globus sensation, hoarseness, pulsatile tinnitus, syncope, transient ischemic attack, or focal neurologic deficits.

Physical examination

Body mass index was 27.8 kg/m. Oropharyngeal examination revealed no visible mass, tonsillar hypertrophy, or obvious pulsatile posterior pharyngeal wall bulge at rest. Otoscopy demonstrated normal tympanic membranes bilaterally. There was no cervical lymphadenopathy. Cranial nerve and complete neurologic examinations were intact. The external auditory canals were clear bilaterally without erythema, edema, or discharge.

Imaging

**Figure 1:**
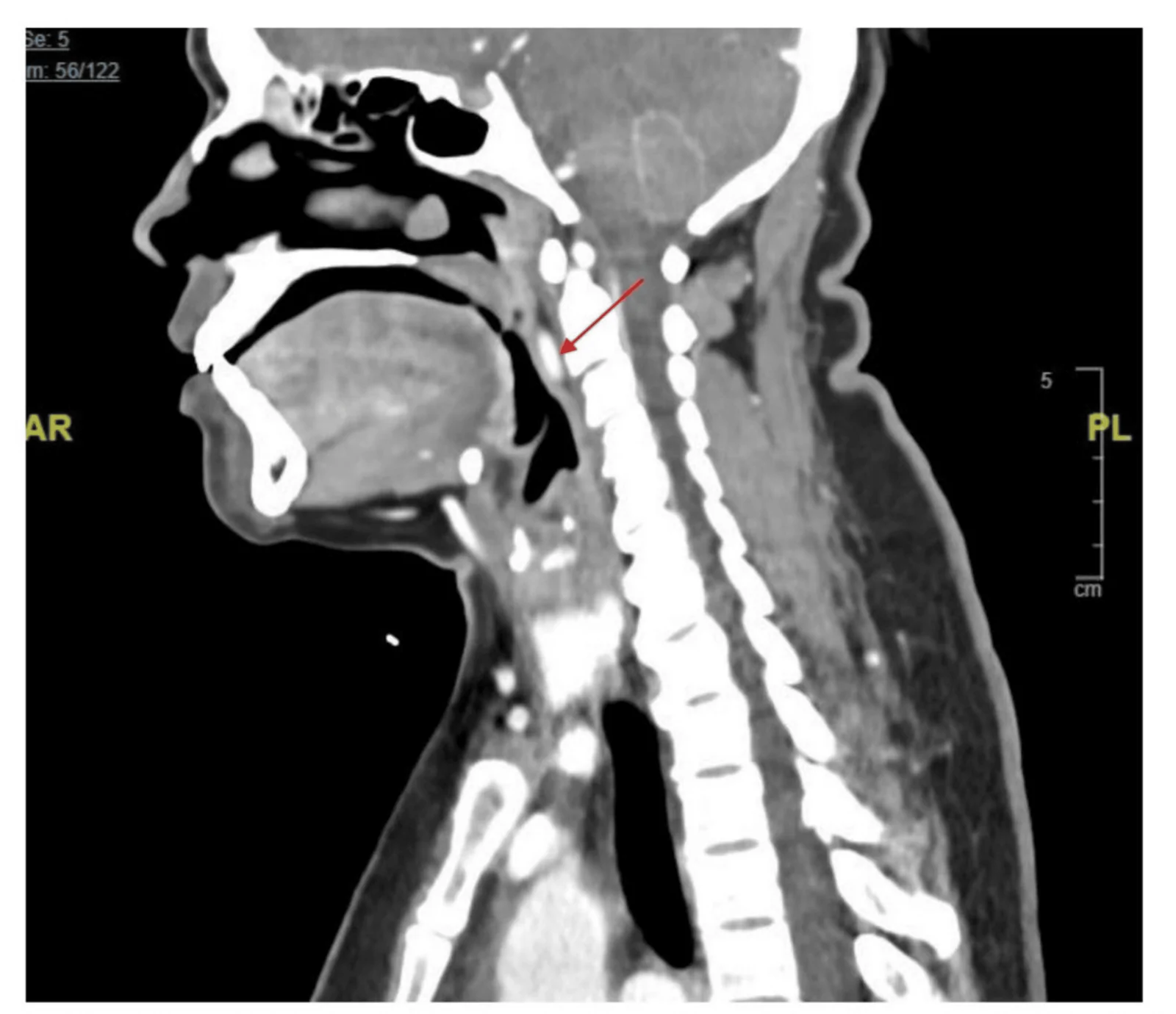
Sagittal CT of the neck with intravenous contrast (AR, PL) demonstrating the aberrant right ICA (red arrow) in its retropharyngeal course. The vessel courses posterior to the oropharyngeal and hypopharyngeal lumen and anterior to the cervical vertebral column. The narrow mucosal interval underscores the catastrophic hemorrhagic risk of blind pharyngeal instrumentation. No aneurysm or dissection is identified. AR, anterior right; PL, posterior left; ICA, internal carotid artery.

Computed tomography of the neck with intravenous contrast was performed. Sagittal reconstruction (Figure 1) demonstrated the right ICA coursing in a retropharyngeal position immediately posterior to the pharyngeal mucosa, approaching within approximately 1 mm of the mucosal surface at its closest point.

**Figure 2:**
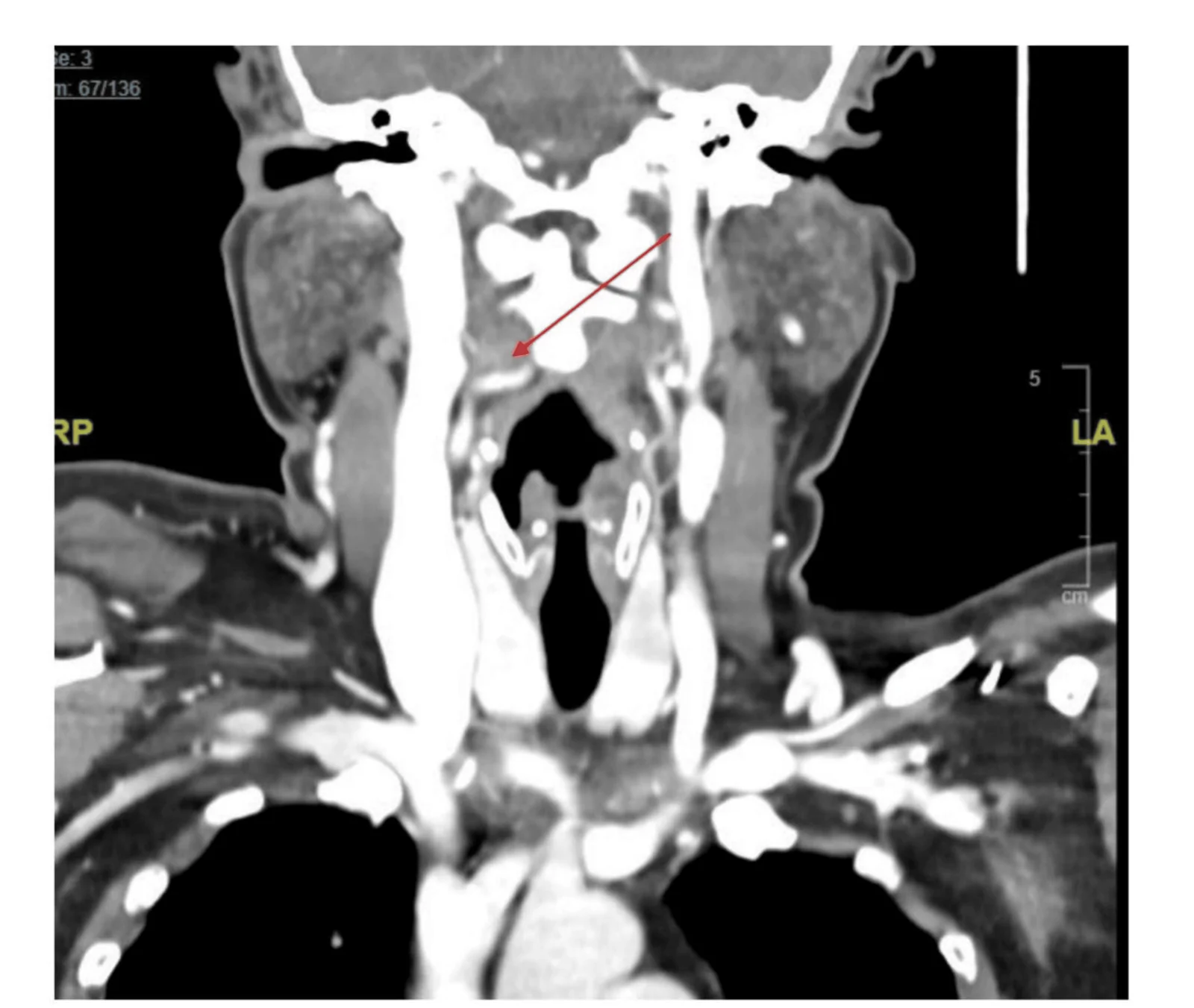
Coronal CT of the neck with intravenous contrast (RP, LA) demonstrating medial displacement of the right ICA (red arrow) into the retropharyngeal space. Unilateral displacement without midline crossing is consistent with Pfeiffer-Ridder Type II classification [1]. RP, right posterior; LA, left anterior; ICA, internal carotid artery.

Coronal reconstruction (Figure 2) confirmed unilateral medial displacement of the right ICA into the retropharyngeal space without midline crossing.

**Figure 3:**
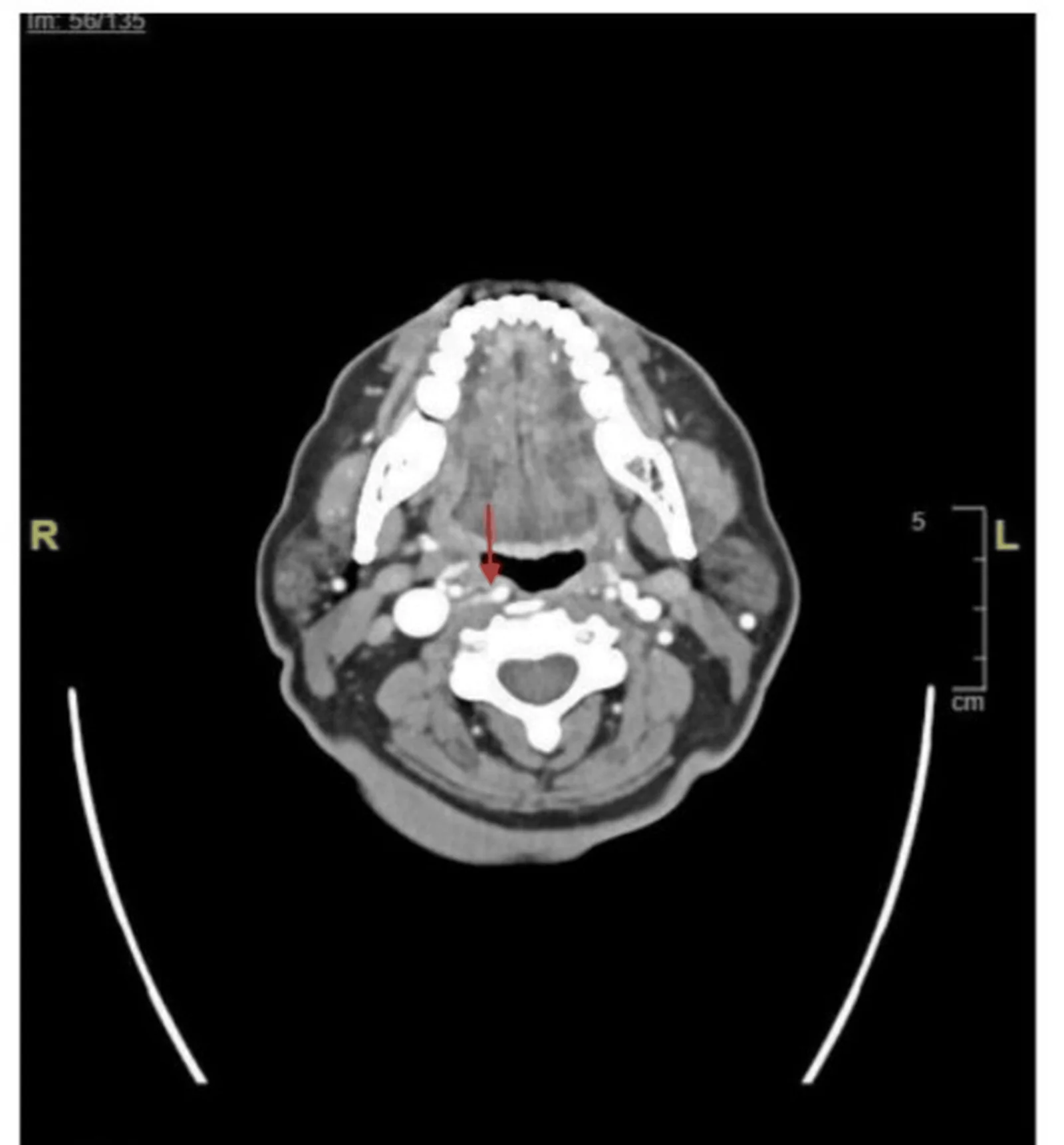
Axial CT images of the neck with intravenous contrast (R, L) demonstrating medial displacement of the right ICA (red arrow) into the retropharyngeal space. Sequential axial images show the right ICA coursing along the posterior pharyngeal wall in closer proximity to the pharyngeal mucosa than the contralateral vessel. R, right; L, left; ICA, internal carotid artery.

Axial reconstruction (Figure 3) further demonstrated asymmetric medialization of the right ICA along the posterior pharyngeal wall, with the vessel positioned closer to the pharyngeal mucosa than the contralateral ICA and without crossing the midline. The left ICA maintained a more typical lateral cervical course. No aneurysm, dissection, mural thrombus, or hemodynamically significant stenosis was identified. Incidental bilateral parotid gland enlargement was noted without focal mass or inflammatory changes.

Endoscopic findings

**Figure 4:**
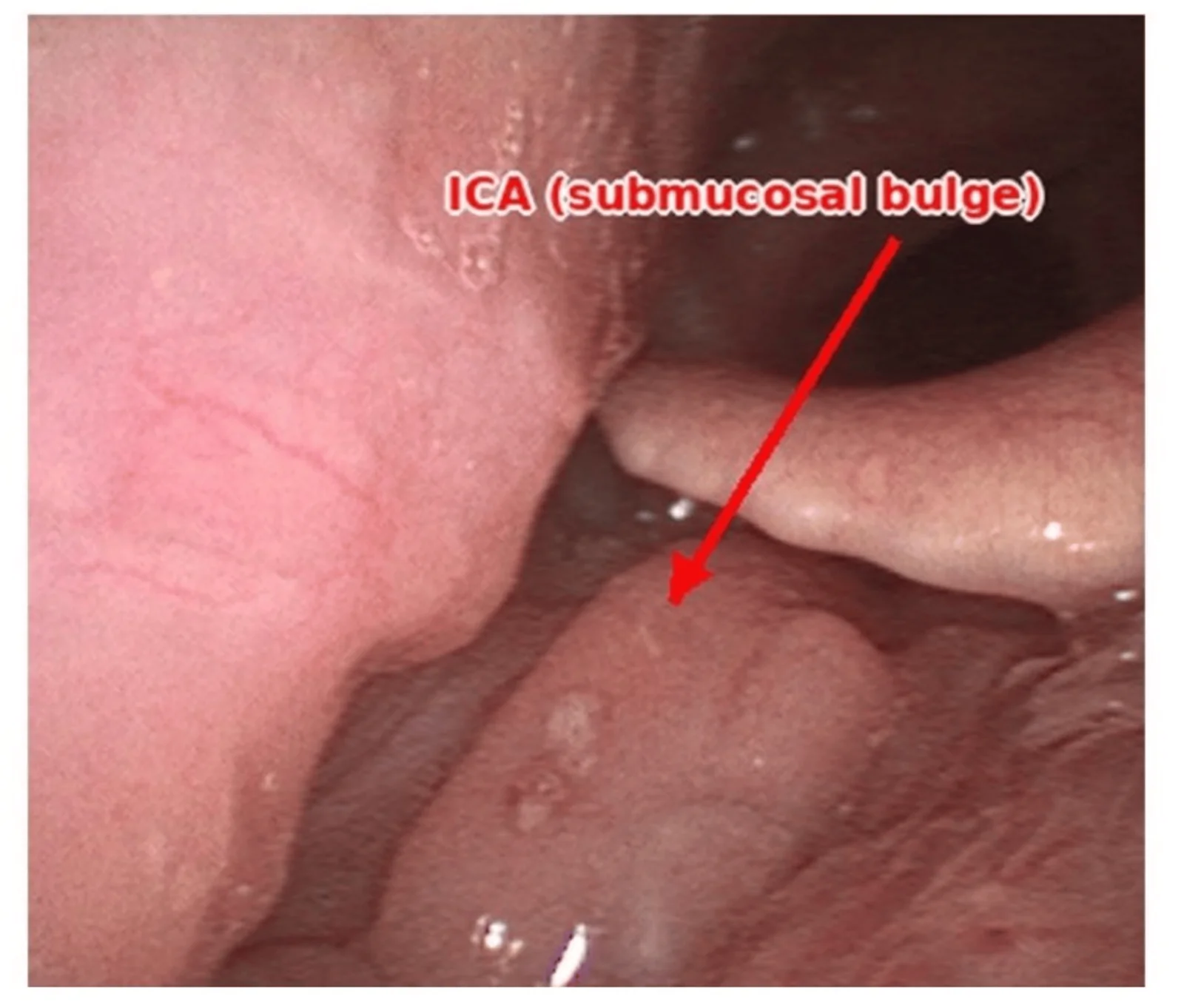
Office flexible fiberoptic laryngoscopy demonstrating right-sided pulsatile submucosal bulging of the posterior pharyngeal wall (red arrow). The finding is pathognomonic for an underlying vascular structure. Laryngopharyngeal reflux stigmata are absent. ICA, internal carotid artery.

Flexible fiberoptic laryngoscopy demonstrated right-sided submucosal bulging of the posterior pharyngeal wall with prominent pulsations synchronous with the cardiac cycle-a finding pathognomonic for an underlying vascular structure (Figure 4). No visible laryngoscopic findings typically associated with laryngopharyngeal reflux (LPR) were observed. No biopsy, aspiration, or direct instrumentation of the bulge was performed, given its vascular appearance.

Vascular evaluation

**Figure 5:**
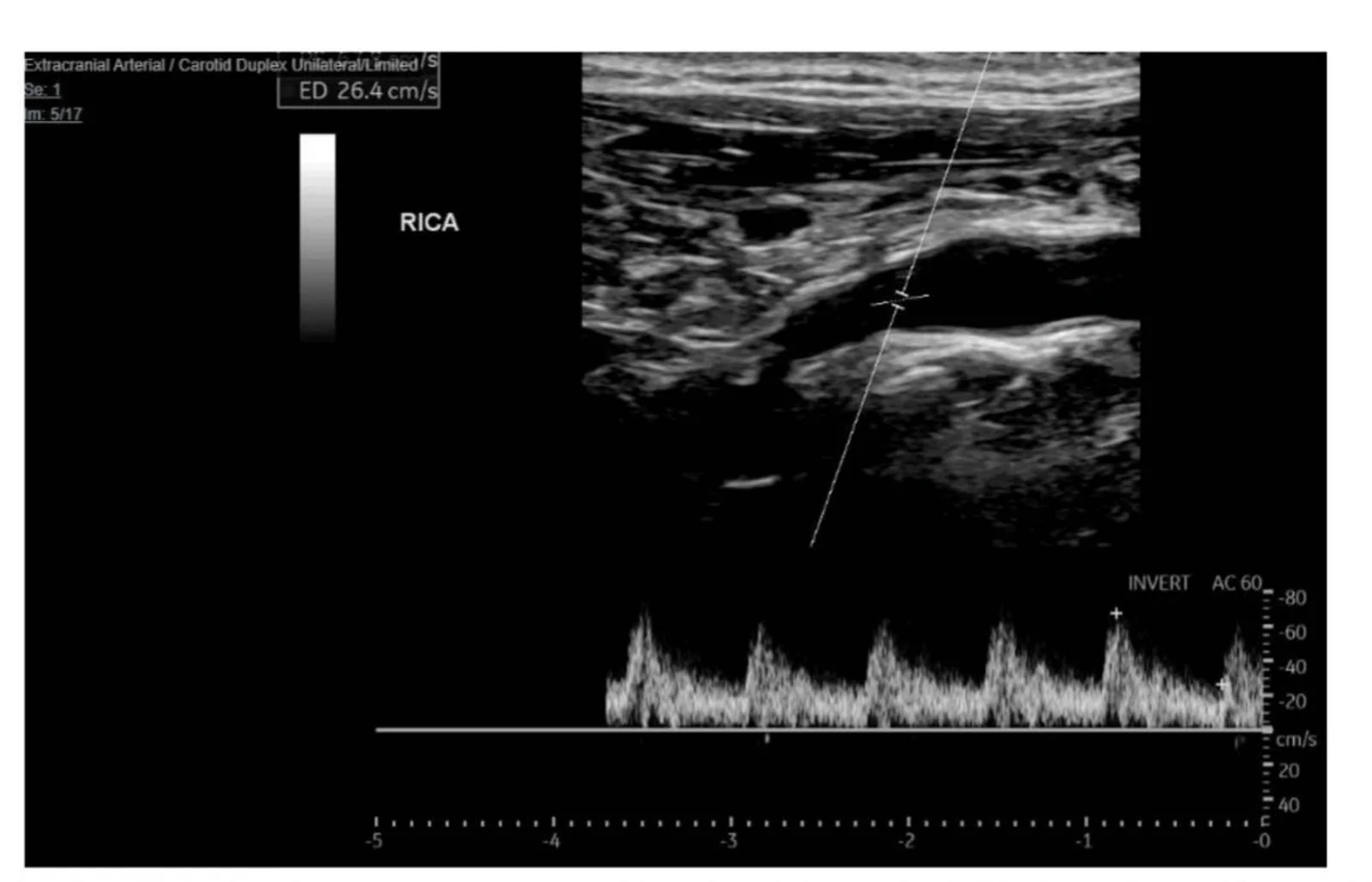
Carotid duplex ultrasonography, spectral Doppler of the distal right internal carotid artery, distal segment (RICA-D). Normal waveform morphology without post-stenotic turbulence or aliasing. The proximal-to-distal velocity gradient is within acceptable physiologic variation. No hemodynamically significant stenosis is identified.

Vascular surgery consultation was obtained. Carotid duplex ultrasound was performed as an extracranial arterial evaluation. Spectral Doppler analysis of the proximal right ICA (RICA; Figure 5) demonstrated an end-diastolic velocity (EDV) of 26.4 cm/s with a low-resistance waveform pattern and no spectral broadening. No stenosis was identified in either carotid artery, and vertebral artery flow was confirmed antegrade bilaterally. In the absence of aneurysm, dissection, significant stenosis, or neurologic symptoms, no surgical or endovascular intervention was recommended.

## Discussion

Epidemiology and anatomical classification

The prevalence of clinically relevant ICA tortuosity or retropharyngeal displacement is difficult to establish precisely. Paulsen et al. reported that approximately 7% of cadaveric specimens demonstrated significant ICA looping with pharyngeal wall contact [2], while radiologic series show considerably variable prevalence depending on imaging modality and population studied, ranging from approximately 2.6% with severe medial displacement to 12.3% with any degree of medialization on cervical MRI [10], and up to 4.5% incidence on contrast-enhanced neck CT angiography [11]. The Pfeiffer-Ridder classification (2008) stratifies the anomaly into four types: Type I (elongation without retropharyngeal extension), Type II (retropharyngeal, no midline crossing), Type III (midline crossing), and Type IV (contralateral extension) [1,12]. The present case represents a Type II variant, confirmed on coronal CT reconstruction (Figure 2), demonstrating unilateral medial displacement of the right ICA without midline crossing.

Embryology and pathogenesis

The ICA develops from the third aortic arch and dorsal aorta during weeks four through seven of embryogenesis. Congenital medial displacement may result from persistence of embryologic anastomoses or failure of normal vessel straightening during cephalic flexure [2,13]. Acquired causes predominate in adults, where progressive arterial elongation secondary to smooth muscle loss, elastic fiber degradation, and intimal thickening from atherosclerosis leads to vessel buckling and retropharyngeal herniation [4,14]. The convergence of hypertension, hyperlipidemia, type 2 diabetes mellitus, and overweight status in this patient is consistent with the established acquired risk factor profile.

Clinical presentation and diagnostic delay

Common presentations of retropharyngeal ICA include pulsatile posterior pharyngeal mass (most specific), dysphagia, globus sensation, throat pain, and referred otalgia [7,8]. Pulsatile tinnitus has been reported when the aberrant vessel approximates the Eustachian tube orifice [15]. Referred otalgia in the present case most plausibly arose via two cranial nerve pathways: (i) Jacobson’s nerve (tympanic branch of CN IX), mediating somatic afferents from the oropharynx to the middle ear, and (ii) Arnold’s nerve (auricular branch of CN X), mediating referred sensation from the hypopharynx to the posterior auricle and external auditory canal [16].

The three-year diagnostic delay in this case reflects a well-recognized pattern. Negative ear cultures and failure of topical otologic treatment should prompt reconsideration of referred otalgia mechanisms. Failure of adequate-dose proton pump inhibitor (PPI) and H2-receptor antagonist therapy for two months constitutes strong evidence against LPR as the primary diagnosis and should prompt cross-sectional imaging before further empirical treatment [9,17].

Imaging and laryngoscopic diagnosis

Contrast-enhanced CT of the neck is the recommended first-line imaging modality [11,18]. The sagittal reconstruction (Figure 1) best demonstrates the retropharyngeal course and critical mucosal proximity, while the coronal reconstruction (Figure 2) confirms the Pfeiffer-Ridder type and excludes midline crossing. Carotid duplex is essential for hemodynamic assessment and excludes stenosis; CT angiography or magnetic resonance angiography (MRA) provides superior vascular resolution for pre-interventional planning; digital subtraction angiography (DSA) is reserved for pre-interventional roadmapping.

Flexible laryngoscopy characteristically reveals a smooth, pulsatile submucosal bulge on the posterior pharyngeal wall, synchronous with the cardiac cycle [8]. This finding distinguishes the lesion from an abscess, lipoma, or neoplasm and should preclude any biopsy or instrumentation. The combination of characteristic laryngoscopic appearance and confirmatory CT imaging is sufficient for diagnosis in the absence of vascular pathology requiring intervention.

Procedural safety

The foremost clinical implication of retropharyngeal ICA is the risk of catastrophic hemorrhage during routine pharyngeal and airway procedures. Published reports document life-threatening or fatal ICA injury during the following [3,5,6,19]: (i) tonsillectomy and adenotonsillectomy; (ii) peritonsillar abscess drainage (needle aspiration or incision and drainage); (iii) transoral biopsy of posterior pharyngeal wall or tonsillar fossa lesions; (iv) adenoidectomy, particularly with blind curettage technique; (v) orotracheal intubation, especially difficult or blind laryngoscopy; (vi) nasopharyngoscopy or pharyngoscopy with tissue sampling; (vii) transoral robotic surgery (TORS) of the oropharynx.

Among the iatrogenic causes of retropharyngeal internal carotid artery injury, tonsillectomy and adenotonsillectomy are the most commonly implicated procedures. Peritonsillar abscess drainage, whether by needle aspiration or incision and drainage, also carries significant risk, as does transoral biopsy of posterior pharyngeal wall or tonsillar fossa lesions. Adenoidectomy poses particular concern when performed using the blind curettage technique, while orotracheal intubation, especially when difficult or performed under blind laryngoscopy, represents another important risk. Additional procedural risks include nasopharyngoscopy or pharyngoscopy with tissue sampling, as well as transoral robotic surgery (TORS) of the oropharynx.

The proximity of the vessel to the mucosal surface, as demonstrated on sagittal CT (Figure 1), means a superficial mucosal incision or needle puncture could violate the arterial wall. ICA hemorrhage is pulsatile, high-pressure, and rapidly exsanguinating; it cannot be reliably tamponaded by pharyngeal packing. Endovascular rescue (balloon occlusion or stent-graft) may be required emergently. The clinical axiom “never instrument a pulsatile pharyngeal mass without prior cross-sectional imaging” must be reinforced across otolaryngology, anesthesiology, emergency medicine, and dentistry.

Management

When conservative management is deemed appropriate, a structured and systematic approach is essential. Initial workup should include contrast-enhanced CT of the neck to characterize the vessel course and its proximity to the pharyngeal mucosa, complemented by carotid duplex ultrasonography to assess hemodynamics, including end-diastolic velocity, peak systolic velocity, and waveform morphology. Vascular surgery consultation should be obtained, and cardiovascular risk factors such as blood pressure, lipids, glucose, and BMI should be optimized. From a safety standpoint, a prominent electronic medical record alert visible across all encounter types is critical, alongside written notification to the patient’s primary care physician, dentist, and anesthesiology service; this is not standard of care but is proposed by the authors. The patient should also carry a written medical alert card specifying the anomaly and any procedural contraindications. Surveillance imaging is recommended at 12 to 24 months or sooner should symptoms change, according to the cases previously reviewed.

Surgical or endovascular intervention is reserved for patients with associated vascular pathology such as aneurysm, pseudoaneurysm, or dissection, as well as those with progressive cerebrovascular symptoms or severe refractory symptoms. Reported approaches include carotid transposition, vessel ligation with extracranial-intracranial bypass, and endovascular stent-graft placement.

To consolidate the key clinical features discussed throughout this report, we have compiled the relevant data into a single comprehensive summary table (Table 1). This table outlines the prevalence and risk factors associated with retropharyngeal internal carotid artery disease, alongside its characteristic symptom profile, diagnostic red flags, and recommended imaging strategy encompassing both first-line and adjunctive modalities. It further details the expected laryngoscopic findings, the Pfeiffer-Ridder anatomical classification with the present case designated as Type II, and the procedural contexts carrying the highest risk of inadvertent vascular injury. Finally, the table summarizes the management framework, distinguishing conservative from interventional approaches, and enumerates the patient safety measures warranted upon diagnosis. Together, these elements provide a structured reference to guide clinicians in the recognition, workup, and safe management of this rare but potentially catastrophic anatomical variant.

**Table 1:** Key clinical learning points. ICA, internal carotid artery; LPR, laryngopharyngeal reflux; CT, computed tomography; IV, intravenous; MRA, magnetic resonance angiography; DSA, digital subtraction angiography; EMR, electronic medical record; PCP, primary care physician.

Category	Details
Prevalence	Rare; approximately 7% ICA pharyngeal looping in cadaveric studies [2]; neck imaging prevalence ranges from approximately 2.6% (severe medialization) to 12.3% (any medialization) on cervical MRI, and up to 4.5% on contrast-enhanced CT angiography, depending on modality and population [10,11]
Risk factors	Hypertension, hyperlipidemia, type 2 diabetes mellitus, overweight, advancing age, atherosclerosis [4,14]
Symptoms	Unilateral otalgia, throat burning, globus, dysphagia, pulsatile tinnitus; mimics otitis externa, LPR, or eustachian tube dysfunction [7,8,15]
Diagnostic red flags	Unilateral pulsatile posterior pharyngeal mass; failure of empirical treatment; food-triggered unilateral throat pain [9,17]
First-line imaging	CT neck with IV contrast: vessel course, mucosal proximity in mm, aneurysm, dissection, stenosis [11,18]
Additional imaging	CT angiography or MRA for pre-interventional planning; carotid duplex for hemodynamics; DSA for roadmapping [11,18]
Laryngoscopy	Pulsatile submucosal posterior pharyngeal bulge synchronous with cardiac cycle; no biopsy or instrumentation [8]
Classification	Pfeiffer-Ridder Type I-IV; present case = Type II (retropharyngeal, unilateral, no midline crossing) [1,12]
High-risk procedures	Tonsillectomy, peritonsillar incision and drainage, transoral biopsy, adenoidectomy, blind intubation, transoral robotic surgery [3,5,20]
Management	Conservative if no aneurysm, dissection, stenosis, or neurologic compromise; cardiovascular risk factor optimization
Patient safety	Electronic medical record alert; written notification to primary care physician, dentist, and anesthesiology; patient-carried medical alert card

Table 2 provides a comparative summary of previously reported cases and series involving retropharyngeal or aberrant internal carotid artery. The present case contributes a diagnostically challenging example. Conservative management was instituted with appropriate safety notifications, and the patient remained neurologically intact at six-month follow-up. These previously reported cases support maintaining a high index of suspicion for retropharyngeal internal carotid artery in patients with refractory unilateral oropharyngeal symptoms and highlight the critical role of cross-sectional imaging prior to any invasive pharyngeal instrumentation.

**Table 2:** Summary of previously reported cases and series. ICA, internal carotid artery; LPR, laryngopharyngeal reflux; CT, computed tomography; MRI, magnetic resonance imaging; MRA, magnetic resonance angiography; ENT, ear, nose, and throat; N/A, not applicable.

Author (year)	Age/sex	Presentation	Side	Diagnosis	Procedural risk	Management	Outcome
Paulsen et al. (2000) [2]	Cadaveric n=50	Anatomic prevalence study	Bilateral 14%	Cadaveric dissection and CT	Tonsillectomy, incision and drainage	N/A	7% ICA touching pharyngeal wall
Deutsch et al. (1995) [5]	6 / M	Pulsatile throat mass	Right	CT and MRI	Tonsillectomy	Observation	Asymptomatic at follow-up
Lukins et al. (2016) [3]	Multi-case review	Dysphagia, globus, pulsatile mass	Various	CT angiography	Multiple ENT procedures	Conservative majority	Hemorrhage in 2 cases
Pfeiffer & Ridder (2008) [1]	Series n=18	Dysphagia, otalgia, throat pain	Various	CT/MRI; Type I-IV	All pharyngeal procedures	Conservative vs surgical	No hemorrhage when recognized
Present case (2026)	54 / F	3-year otalgia, burning throat, submandibular pain; mimic of LPR and otitis externa	Right	CT neck, laryngoscopy, duplex	Tonsillectomy, incision and drainage, biopsy, intubation	Conservative; notification initiated	Stable at 6 months; no neurologic events

## Conclusions

Retropharyngeal internal carotid artery is a rare but clinically critical vascular variant that may present as chronic, treatment-refractory unilateral otalgia and throat pain, closely mimicking otitis externa and laryngopharyngeal reflux. A diagnostic challenge, as in this case, illustrates the importance of considering vascular etiologies when symptoms are strictly unilateral and fail to respond to appropriate empirical treatment. Sagittal and coronal contrast-enhanced CT of the neck demonstrated retropharyngeal vessel course and critical mucosal proximity of approximately 1 mm. Carotid duplex confirmed the absence of hemodynamically significant stenosis, supporting conservative management. Flexible laryngoscopy demonstrating pulsatile submucosal bulging is pathognomonic and should prompt immediate imaging evaluation without biopsy. Recognition of this anomaly across specialties is essential to prevent potentially fatal hemorrhage during routine pharyngeal procedures. Especially before biopsy, drainage, tonsillar procedures, or difficult airway manipulation.
